# Microarray and pathway analysis of two COMMA-Dβ derived clones reveal important differences relevant to their developmental capacity *in-vivo*

**DOI:** 10.18632/oncotarget.26655

**Published:** 2019-03-15

**Authors:** Jabril R. Johnson, Corinne A. Boulanger, Tamaro Hudson, Evan Savage, Gilbert H. Smith

**Affiliations:** ^1^ Mammary Stem Cell Biology Section, Basic Research Laboratory, Center for Cancer Research, National Cancer Institute, Bethesda, MD 20892, USA; ^2^ Howard University Cancer Center, Washington, DC 20059, USA; ^3^ Department of Pharmacology, College of Medicine, Washington, DC 20059, USA; ^4^ Genome Explorations, Division of Compass Laboratory Services, Memphis, TN 38105, USA

**Keywords:** breast cancer, stem cell, mammary gland, microarray, genetic expression

## Abstract

Microarray technologies were used to analyze transcriptomes from Comma-Dβ and clonal derivatives, SP3 (Lobule-competent) and NSP2 (Lobule-incompetent), during different mouse mammary growth phases: *in-vitro*, *in-vivo* 5-weeks, and *in-vivo* 12-weeks. A differentially expressed gene (DEG) algorithm was used to enrich for genes associated with cellular proliferation, differentiation, cell cycle regulation, and carcinogenesis. A pairwise comparison analysis, of SP3 vs. NSP2 *in-vitro*, revealed a total of 45 DEGs significantly up-regulated in SP3. Of the 45 DEGs, only Ccnd1 (Cyclin D1), Id2 (Inhibitor of DNA binding 2) and Sox9 (SRY Box 9) were identified to be associated with cellular proliferation, regulation of G1/S mitotic cell cycle, mammary gland and alveolar development in SP3. During the regenerative growth phase, *in-vivo* 5-weeks, we identified a total of 545 DEGs. 308 DEGs, of the 545 DEGs, were significantly up-regulated and 237 DEGs were significantly down-regulated in SP3 vs. NSP2. In addition, we identified 9 DEGs significantly up-regulated, within SP3's cell cycle pathway and a persistent overexpression of Cyclin D1, Id2, and Sox9, consistent with our *in-vitro* study. During the maintenance phase, *in-vivo* 12-weeks, we identified 407 DEGs. Of these, 336 DEGs were up-regulated, and 71 were down-regulated in SP3 vs. NSP2. Our data shows 15 DEGs significantly up-regulated, simultaneously, affecting 8 signal transducing carcinogenic pathways. In conclusion, increased expression of Cyclin D1, Id2 and Sox9 appear to be important for lobular genesis in SP3. Also, *in-vivo* 12 week displays increase expression of genes and pathways, involved in tumorigenesis.

## INTRODUCTION

In 1996, Smith [1] was the first to demonstrate, on the basis of limiting dilution transplantation studies, that the mouse mammary gland contained three distinct progenitors: lobular, ductal and lobuloalveolar. Later, Wagner and colleagues [2] discovered a parity-identified mammary epithelial subpopulation that was defined as a lobular-restricted progenitor cell. This reporter-marked cell type was found in the luminal cell compartment of ducts after transplantation but could not produce either ducts or alveoli by itself after cell sorting (unpublished data). Although previous studies demonstrated the presence of these progenitors, their phenotypic characteristics were not known until now. Flow cytometry (FACS) cell sorting followed by transplantation has allowed the phenotypic and functional characterization of progenitor and differentiated cells in mouse mammary glands and human breast cells [3, 4, 5, 6, 7, 8]. However, the distinct lineage-limited progenitors have not been prospectively isolated, expanded *in-vivo* or *in-vitro* or fully characterized. Employing single-cell cloning, distinct mammary progenitor populations were isolated. The COMMA-D (CD) cell line was initially derived from mid-pregnant Balb/C mouse mammary glands [9]. This cell line was unique in that transplantation of cells into the epithelium-free fat pads of syngeneic female mice generated mammary ductal and alveolar structures Including myoepithelium. The CD cell line harbors two distinct p53 mutations: (1) a G-to-C transversion resulting in the substitution of tryptophan for cysteine at codon 138 and (2) a deletion of the first 21 nucleotides of exon 5 resulting in deletion of codons 123 to 129 [10]. The experiments in this study were performed using COMMA-D cell line engineered to express β-galactosidase (CDβ) cells. CDβ cells were derived from the parental CD cells by transduction with Zeg^+^ retrovirus containing a bifunctional LacZ/Neomycin (β-geo) generated in Dr. Soriano's laboratory [11, 12]. Detailed sequence analysis of the *p53* gene in eight different clonal derivatives of the CDβ cell line showed that both mutant alleles were present in each clone, demonstrating that the CD cell line is indeed clonal with respect to the *p53* gene and that each cell expresses two distinctive mutant alleles of *p53* [10]. In addition, each growth-competent clone produced outgrowths, *in-vivo*, comprised of both luminal and basal cell types [13]. As a result of these *p53* mutations, the mammary outgrowths progress to mammary tumors after many months *in-vivo*. Our laboratory [14] previously showed that clones NSP-2, SP-3, and NSP-3 were positive for long-label retaining cells (LRC) and both ER-α and PR-positive luminal cells. This argues that each clone is at least bi-potent, if not multipotent. Here microarray analysis of ductal-limited (NSP-2), alveolar-limited (SP-3) and lobuloalveolar-competent mammary progenitor clones are presented. Our study provides a molecular genetic basis for distinguishing ductal-limited, lobuloalveolar-incompetent clones (NSP-2) and lobuloalveolar-competent (SP-3), from the CDβ cell line.

In summary, key differential expressed genes, were identified to be essential in cell cycling, cell proliferation, and differentiation pathways, in NSP2 and SP3. This supports the observation of specific cellular clone fates and limited outgrowths. Further research could elucidate fundamental underlying mechanisms that are essential for tumorigenesis in mouse mammary gland.

## RESULTS

### Confirmation that NSP2 is unable to respond to pregnancy signals and form functional acini

**Figure 1:**
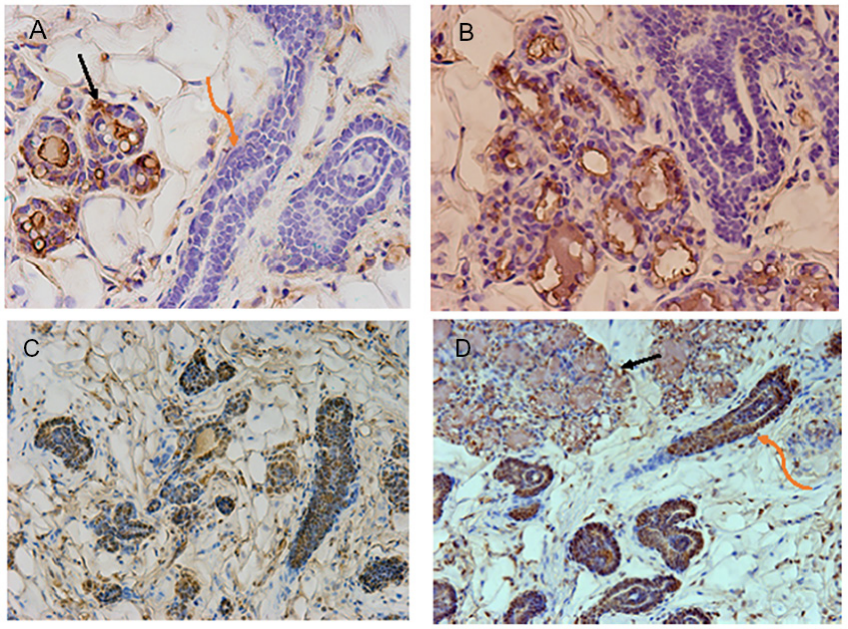
Images shown are immunohistochemical confirmation that NSP2 is unable to respond to pregnancy signals and form functional acini Figure 1 **(A, B)** shows sections stained with anti-total casein. The black arrow indicates alveolar structures formed by normal Balb/C mammary epithelial cells (MEC). The orange arrow indicates NSP2 structures. Casein positive staining is brown precipitate. The NSP2 cells were distinguished from the normal Balb/C by the expression of beta-galactosidase [13]. Figure 1 **(C, D)** shows similar sections from the chimeric outgrowth stained for RANKL (brown). The black arrow indicates alveolar structures. The orange arrow indicates NSP2 structures. The sections were produced from chimeric outgrowths in hosts at parturition.

It was imperative to demonstrate that NSP2 cells would not respond to pregnancy by forming secretory lobules as reported earlier [13]. To test this, NSP2 cells were mixed with Balb/C mammary epithelial cells (50K/50K) and inoculated into the epithelium-cleared mammary fat pads of 4week-old Nu/Nu female mice. These hosts were subsequently bred and allowed to proceed to parturition. At parturition, the outgrowths present in the implanted fat pads were collected fixed and stained for the presence of functional secretory alveoli and the presence of milk proteins and gene products known to be important in alveolar development (Figure 1A, 1B). The NSP2 cells were distinguished from the normal Balb/C by the expression of beta-galactosidase and formed only ductal structures in the full-term pregnant females.

On the other hand, the unmarked normal Balb/C cells formed alveolar structures and secreted milk proteins. As reported earlier [14] NSP2 cells were positively stained for ER and progesterone receptors. However, they were negative for milk protein production even though they were strongly positive for RANKL, a factor strongly promoted by progesterone in normal mammary epithelium (Figure 1C, 1D).

### Differential expressed gene (DEG) variations in COMMA-Dβ Geo clonal derivatives: Lobular Competent (SP3) and Lobular incompetent (NSP2)

The clones (40K cells) were injected into epithelium cleared mammary fat pads of 3-week-old Nu/Nu female mice and were harvested at 5 weeks and at 12 weeks to analyze the transcriptome of growing (5 weeks) and mature (12 weeks) outgrowths. The purpose here was to examine the genes expressed during an active growth phase (5 weeks) and those expressed after active growth had largely ceased (12 weeks). These were compared to those genes expressed by the respective clones during growth *in-vitro* where no contribution from the *in-vivo* microenvironment was present. Comma-Dβ Geo and Balb/C were used as both *in-vitro* and *in-vivo* controls.

**Figure 2:**
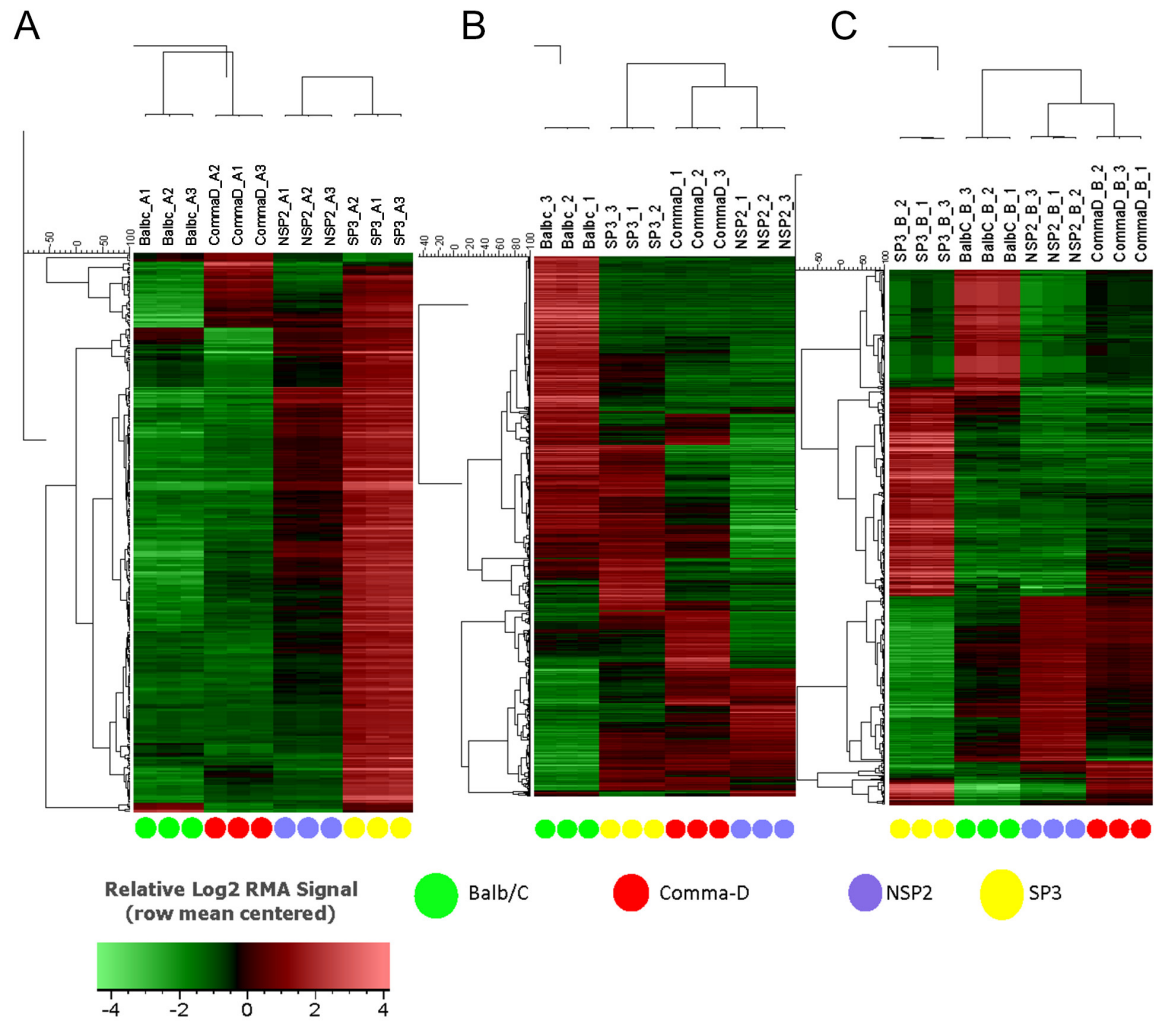
Hierarchical clustering of Balb/C, Comma-Dβ, NSP2, and SP3 cell line transcriptomes All transcript clusters from the MoGene 1.0 ST array were subjected to hierarchical clustering using the UPGMA algorithm based on Pearson correlation metric without any statistical filtering of transcript clusters. Selected cell line samples were analyzed in triplicates for **(A)** *in-vitro*, **(B)** *in-vivo* 5 weeks and **(C)** *in-vivo* 12 weeks.

**Figure 3:**
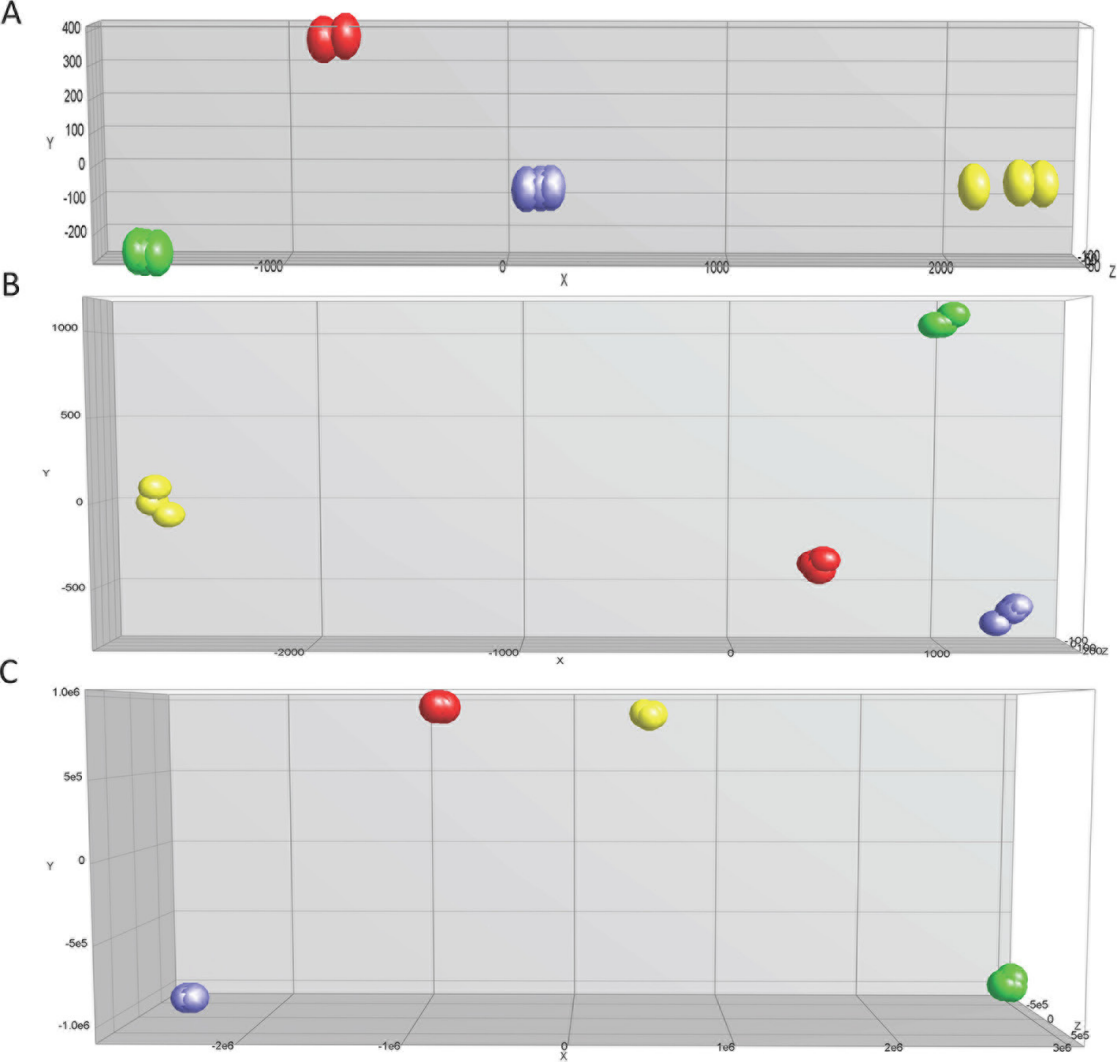
Principal component analysis for expression profile arrays, *in-vitro*, *in-vivo* 5 and 12 weeks The three-dimensional diagram identifies four principal components (PC) that are separated by differential expression gene (DEG) profiles of the selected cell lines analyzed. Green, Red, Blue and Yellow circles represent Balb/C, Comma-Dβ, NSP2, and SP3, respectively. Selected cell line samples were analyzed in triplicates for **(A)** *in-vitro*, **(B)** *in-vivo* 5 weeks and **(C)** *in-vivo* 12 weeks.

Hierarchal unsupervised clusters for *in-vitro*, *in-vivo* 5 and 12 weeks (Figure 2), showed gene probes of total DEGs, up-regulated and down-regulated, analyzed in selected cell lines. Differential gene expression variation was identified using gene expression ratio. (Expression ratio= log2|Fold Change| Balb/C vs. COMMA-Dβ; log2|Fold Change| NSP2 vs. COMMA-Dβ; log2|Fold Change| SP3 vs. COMMA-Dβ; log2|Fold Change| SP3 vs. NSP2. |Fold Change| ≥3 and False Discovery Rate (FDR) adjusted P<.01). Utilizing gene expression ratio, *in-vitro* analysis identified a total of 355 DEGs. Of the 355 DEGs, 45 DEGs were up-regulated and 0 DEGs were down-regulated in SP3 vs. NSP2 when compared to the control groups (Balb/C vs. COMMA-Dβ, NSP2 vs. COMMA-Dβ and SP3 vs. COMMA-Dβ) (Table 1). The 5-week analysis identified 545 DEGs. Of the 545 DEGs, 308 DEGs were up-regulated, and 237 DEGs were down-regulated in SP3 vs. NSP2, compared to the control groups (Table 2). The 12-week analysis identified 407 DEGs. Of the 407 DEGs, 336 DEGs were up-regulated, and 71 DEGs were down-regulated in SP3 vs. NSP2, compared to the control groups (Table 3). Principal Component (PC) analysis showed great specificity and reproducibility. This determined that the transcriptomes of the experimental groups (NSP2 and SP3) were significantly different from one another, both *in-vivo* and *in-vitro* (Figure 3).

**Table 1:** *In-vitro* analysis: sample comparison groups’ differential gene expression

No. of Sample Groups	Sample Comparison Group	No. of Up-regulated Genes	No. of Down-regulated Genes
**1**	Balb/C/Comma-Dβ	2	37
**2**	NSP2/Comma-Dβ	12	7
**3**	SP3/Comma-Dβ	249	3
**4**	SP3/NSP2	45	0

Up and down-regulation of differential expressed genes (DEGs), of sample comparison groups, *in-vitro*, were compiled and tallied. Balb/C, NSP2 (lobular incompetent) and SP3 (lobular competent) were compared to Comma-Dβ (Control). (P≤.01; FDR adjusted; Log2 Fold Change≥3.0).

**Table 2:** *In-vivo* 5-week analysis: sample comparison groups’ differential gene expression

No. of Sample Groups	Sample Comparison Group	No. of Up-regulated Genes	No. of Down-regulated Genes
**1**	Balb/C/Comma-Dβ	79	53
**2**	NSP2/Comma-Dβ	23	11
**3**	SP3/Comma-Dβ	100	203
**4**	SP3/NSP2	308	237

Up and down-regulation of differential expressed genes (DEGs), of sample comparison groups, *in-vivo 5-weeks*, were compiled and tallied. Balb/C, NSP2 (lobular incompetent) and SP3 (lobular competent) were compared to Comma-Dβ (Control). (P≤.01; FDR adjusted; Log2 Fold Change≥3.0).

**Table 3:** *In-vivo* 12-week analysis: sample comparison groups’ differential gene expression

No. of Sample Groups	Sample Comparison Group	No. of Up-regulated Genes	No. of Down-regulated Genes
**1**	Balb/C/Comma-Dβ	393	283
**2**	NSP2/Comma-Dβ	30	261
**3**	SP3/Comma-Dβ	134	94
**4**	SP3/NSP2	336	71

Up and down-regulation of differential expressed genes (DEGs), of sample comparison groups, *in-vivo 12 weeks*, were compiled and tallied. Balb/C, NSP2 (lobular incompetent) and SP3 (lobular competent) were compared to Comma-Dβ (Control). (P≤.01; FDR adjusted; Log2 Fold Change≥3.0).

### Gene Ontology (GO) enrichment analysis of Differential Expressed Genes (DEGs)

Differentially expressed genes, implicated in lobular competent and incompetent, were analyzed by DAVIDS’ GO for the top 10 probe sets of pathway, cluster, and network enrichment. GO analysis allocates genes to various categories for enrichment. This study explored four GO classifications: biological process (BP), molecular function (MF), cell component (CC) and pathway. To determine whether overlap between the differentially expressed gene list and the GO annotation list, Pearson chi-square test was used.

#### Lobular Competent (SP3) vs. Lobular Incompetent (NSP2), *in-vitro* analysis

**Figure 4:**
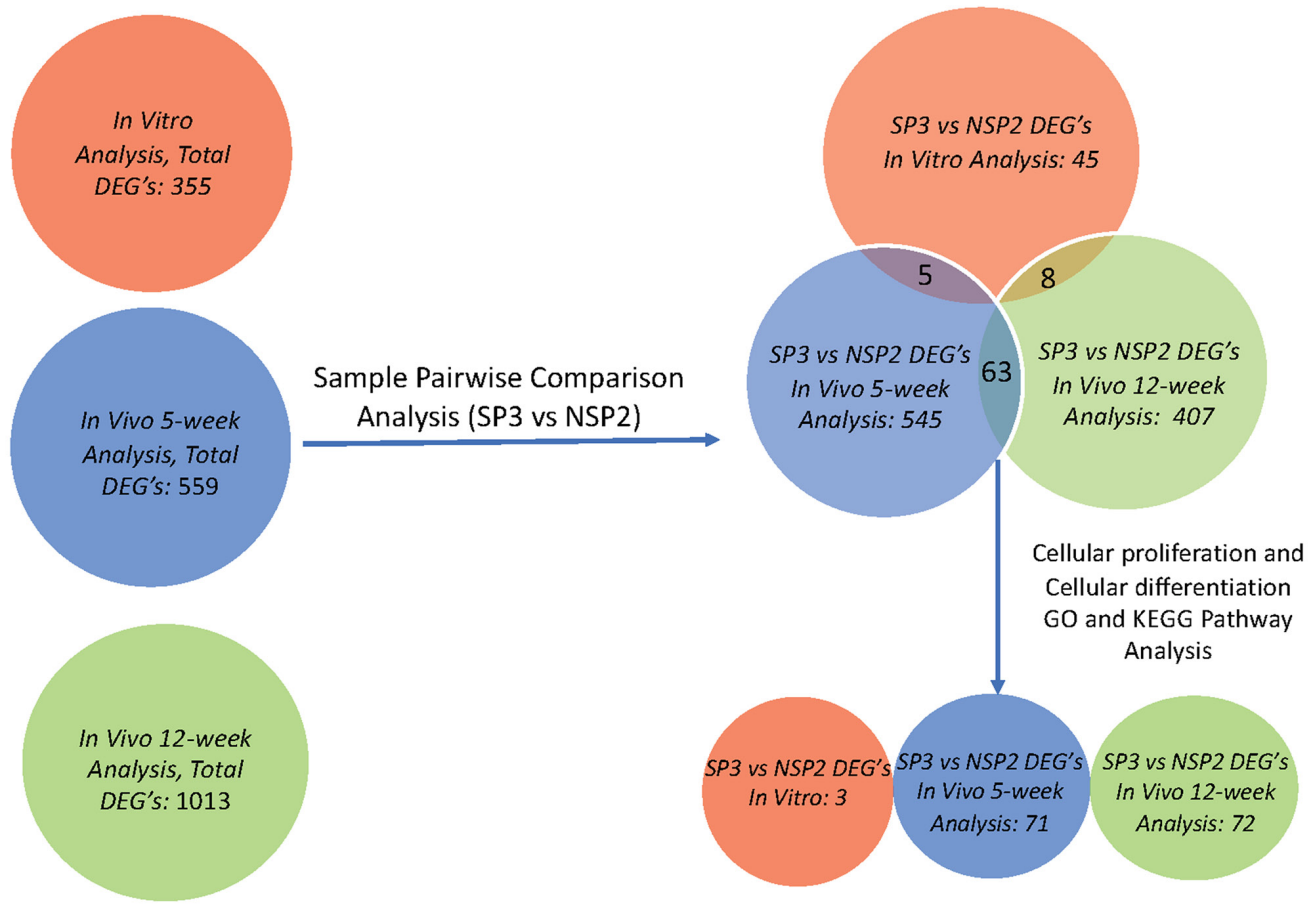
Gene screening algorithm and DEGs intersection of *in-vitro*, *in-vivo* 5 and 12-week analysis Total DEGs were identified utilizing differential gene expression parameter: log2|Fold Change|≥3.0. Total DEGs, for *in-vitro* (Red), *in-vivo* 5 weeks (Blue) and *in-vivo* 12 weeks, was further analyzed using sample pairwise comparison to select and identify significant DEGs from SP3 vs. NSP2. SP3 vs. NSP2, pairwise comparison analysis, of each study, also revealed overlapping common DEGs, through intersection. GO, and KEGG pathway analysis further screened for significant DEGs that clustered in cellular proliferation and cellular differentiation pathways of SP3 vs. NSP2.

**Figure 5:**
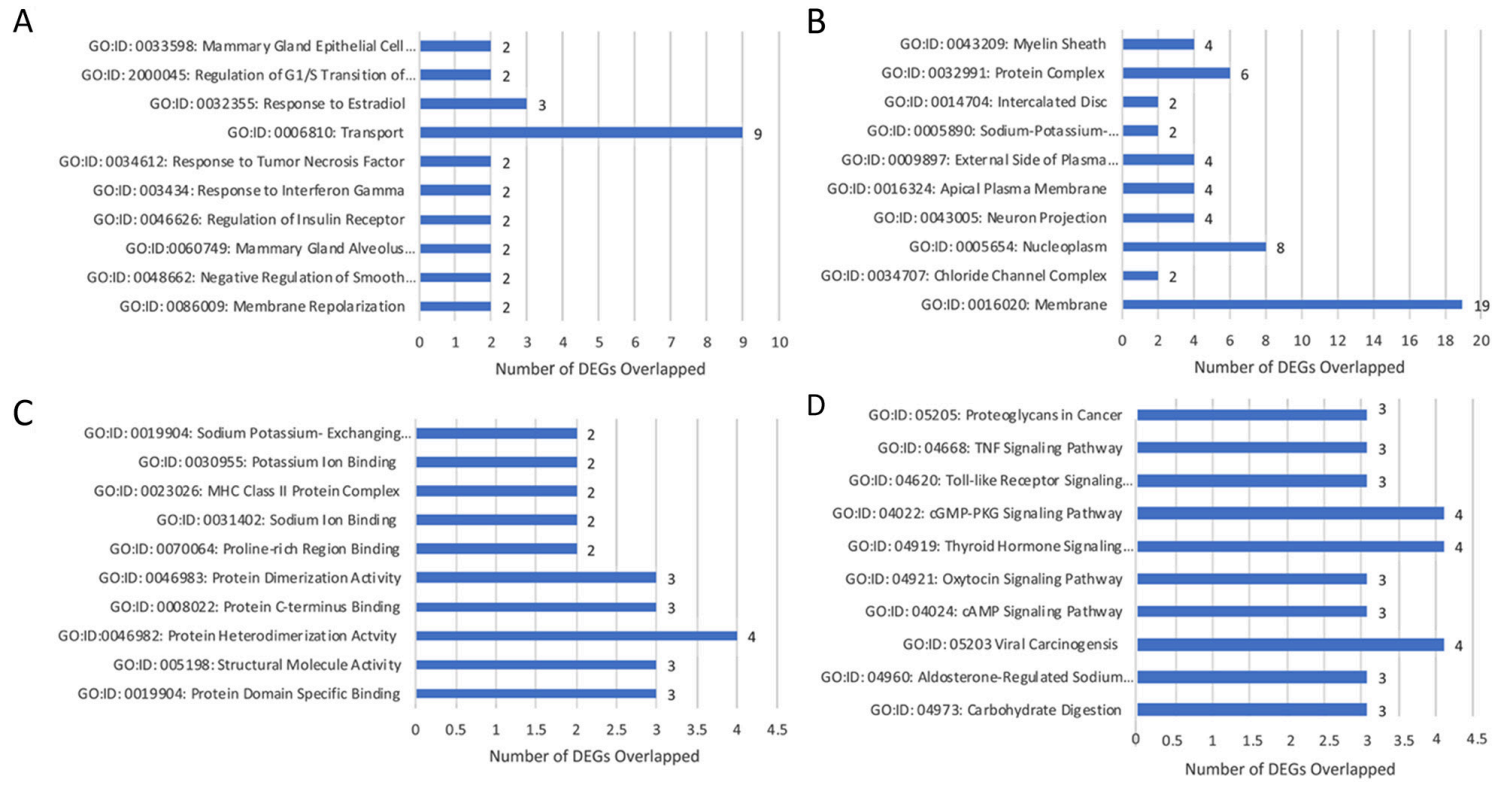
Gene annotation (GO) enrichment analysis for top 10 probe sets, for SP3 vs. NSP2, *in-vitro* Bar graphs display total differential expression genes (DEGs) overlapped and clustered in each GO group within each GO category. The GO covers four categories: **(A)** Biological Processes, **(B)** Cellular Components, **(C)** Molecular Functions and **(D)** Pathway. (P≤0.05; DEG Fold Change ≥3.0). Enrichment of pathway analysis is performed with KEGG.

DEGs were selected using gene selection algorithm (Figure 4). 45 DEGs were referred and allocated to the top 10 each of biological processes; cellular components; and molecular functions. Results of *in-vitro* analysis of the biological processes in lobular competent vs. lobular incompetent clones suggest that 2 of the 45 genes are implicated in mammary gland epithelial cell proliferation, regulation of G1/S transition of mitotic cell cycle, response to estradiol and mammary gland alveolus development (Figure 5A). Of the 45 genes implicated in the cellular component GO category, 23 genes were allocated to membrane development. The remaining differential genes were associated with non-membrane development which included: myelin sheath, protein complex, sodium/potassium-exchanging ATPase complex, neuron projection and chloride channel complex (Figure 5B). Molecular function, in the GO category, revealed 26 genes, 12 of these genes were associated with the enzymatic activity, and 14 were identified in binding activity (Figure 5C). DEGs selected by GO term enrichment analysis showed low P-values, indicating considerable significance (P≤.05, FDR adjusted, Q<.05 was recommended) (Figure 5).

#### Lobular Competent (SP3) vs. Lobular Incompetent (NSP2), *in-vivo* 5-weeks analysis

**Figure 6:**
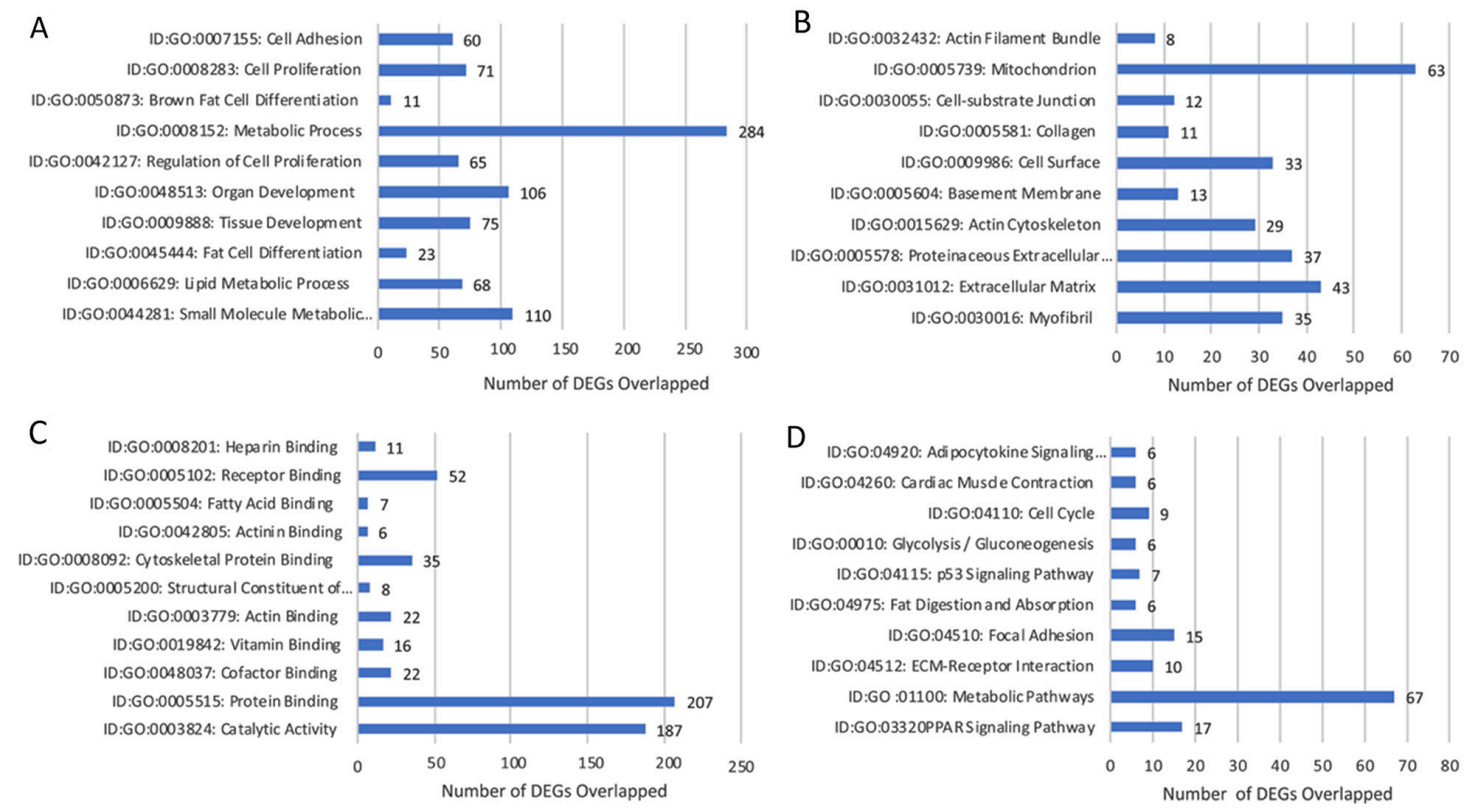
Gene annotation (GO) enrichment analysis for top 10 probe sets, for SP3 vs. NSP2, *in-vivo* 5 weeks Bar graphs display total differential expression genes (DEGs) overlapped and clustered in each GO group within each GO category. The GO covers four categories: **(A)** Biological Processes, **(B)** Cellular Components, **(C)** Molecular Functions and **(D)** Pathway. (P≤0.05; DEG Fold Change ≥3.0). Enrichment of pathway analysis is performed with KEGG.

DEGs were selected using the gene selection algorithm (Figure 4). A total of 545 DEGs were identified for biological processes; cellular components; and molecular functions when comparing all of the data, including the controls. Results of 5-week *in-vivo* analysis of the biological processes in lobular competent (SP3) vs. lobular incompetent (NSP2) revealed 182 genes involved in tissue and organ development. Seventy-one (71) genes were implicated in the regulation of cellular proliferation. In addition, 23 genes were identified in fat cell differentiation, which overlapped with 11 genes specifically involved in brown fat cell differentiation (Figure 6A). GO analysis of cellular component identified 128 genes associated with cellular integrity including actin filament bundle, cell-substrate junction, collagen, actin cytoskeleton, and myofibrils. Those involved with extracellular matrix consisted of 43 genes (Figure 6B). Of the total 545 DEGs identified, the molecular function of 343 genes were involved in the binding activity of proteins, co-factor, vitamin, actin, actinin, fatty acid receptor and heparin (Figure 6C). DEGs selected by GO term enrichment analysis showed low P-values, indicating considerable significance (P≤.05, FDR adjusted, Q<.05 was recommended) (Figure 6).

#### Lobular Competent (SP3) vs. Lobular Incompetent (NSP2), *in-vivo* 12-weeks analysis

**Figure 7:**
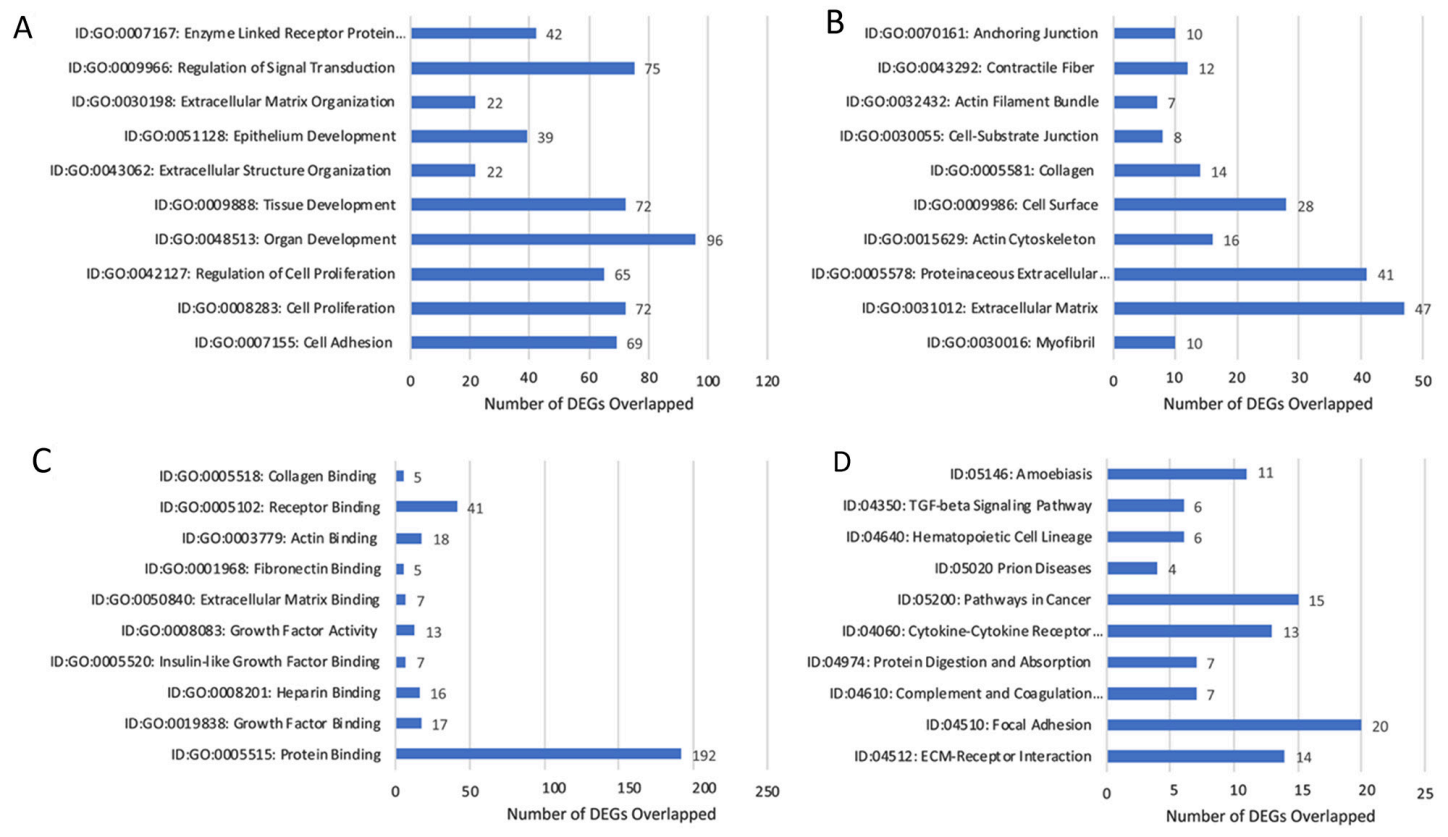
Gene annotation (GO) enrichment analysis for top 10 probe sets, for SP3 vs. NSP2, *in-vivo* 12 weeks Bar graphs display total differential expression genes (DEGs) overlapped and clustered in each GO group within each GO category. The GO covers four categories: **(A)** Biological Processes, **(B)** Cellular Components, **(C)** Molecular Functions and **(D)** Pathway. (P≤0.05; DEG Fold Change ≥3.0). Enrichment of pathway analysis is performed with KEGG.

DEGs were selected using gene selection algorithm (Figure 4). 407 genes were referred to biological processes; cellular component; and molecular functions. GO analysis of biological processes in, lobular competent (SP3) vs. lobular incompetent (NSP2) *in-vivo* 12-weeks, resulted in 168 genes involved in organ and tissue development. Epithelial development and cellular proliferation implicated 111 genes as a necessity in lobular genesis (Figure 7A). GO analysis of cellular component identified 87 genes to be associated with myofibril, actin cytoskeleton, cell surface, collagen and anchoring junction systems (Figure 7B). Of the 321 genes distributed, by the GO analysis to molecular functions, 129 genes were implicated in binding. Specifically, the 129 gene list associated with binding, was comprised of: growth factor binding, heparin binding insulin-like growth factor binding, fibronectin binding, actin binding, receptor binding and collagen binding (Figure 7C). DEGs selected by GO term enrichment analysis showed low P-values, indicating considerable significance (P≤.05, FDR adjusted, Q<.05 was recommended) (Figure 7).

### KEGG pathway analysis

Pathway analysis and enrichment aids in elucidating genes that work in concert to perform and regulate biological functions. KEGG pathway enrichment identified several diverse pathways implicated in the lobular competent vs. the lobular incompetent cell line, *in-vitro*, *in-vivo* 5-weeks and *in-vivo*-12 weeks. The top ten enriched pathways were identified for each analysis.

#### Lobular Competent (SP3) vs. Lobular Incompetent (NSP2), *in-vitro* analysis

The top 10 enriched pathways implicated in the *in-vitro* analysis of SP3 vs. NSP2 included: Proteoglycans in cancer, Tumor necrosis factor (TNF) signaling pathway, Toll-like receptor signaling pathway, cGMP-PKG signaling pathway, Thyroid hormone signaling pathway, Oxytocin signaling pathway, cAMP signaling pathway, Viral carcinogenesis, Aldosterone-regulated sodium reabsorption and Carbohydrate digestion/absorption (Figure 5D). Further analysis of the enriched pathway data identified 2 significant DEGs overlapped in the following pathways: Proteoglycans in cancer, Thyroid hormone signaling pathway, Viral carcinogenesis pathway, and Oxytocin signaling pathway (Figure 5D; P≤.05, FDR adjusted <.05).

#### Lobular Competent (SP3) vs. Lobular Incompetent (NSP2), *in-vivo* 5-weeks analysis

The top 10 enriched pathways implicated in the *in-vivo* 5-week analysis of SP3 vs. NSP2 included: PPAR signaling pathway, Metabolic pathways, ECM-receptor interaction, Focal Adhesion, Fat digestion and absorption, p53 signaling pathway, Glycolysis/Gluconeogenesis, Cell cycle, Cardiac muscle contraction and Adipocytokine signaling pathway (Figure 6D). Investigating significant DEGs that cluster within pathways revealed 9 genes, up-regulated ≥3.0 working in concert, within the cell cycle pathway (Figure 6D; P≤.05, FDR adjusted <.05).

#### Lobular Competent (SP3) vs. Lobular Incompetent (NSP2), *in-vivo* 12-weeks analysis

The top 10 enriched pathways implicated in the *in-vitro* analysis, of SP3 vs. NSP2 included: ECM-receptor interaction, Focal adhesion, Complement and coagulation cascades, Protein digestion and absorption, Cytokine-Cytokine receptor interaction, Pathways in cancer, Prion disease, Hematopoietic cell lineage, TGF-beta signaling pathway and Amoebiasis (Figure 7D). Pathway enrichment data further reveals 15 significantly DEG clusters implicated in pathways in cancer, 6 implicated in the TGF-beta signaling pathway and 13 involved in the Cytokine-Cytokine receptor interaction. Although DEGs do not show overlap between pathways, these specific pathways were identified to be significantly associated with SP3 development (Figure 7D; P≤.05, FDR adjusted <.05).

### Differentially Expressed Genes (DEGs) associated with cellular proliferation and gland development in Lobular Competent (SP3) vs. Lobular Incompetent (NSP2)

**Figure 8:**
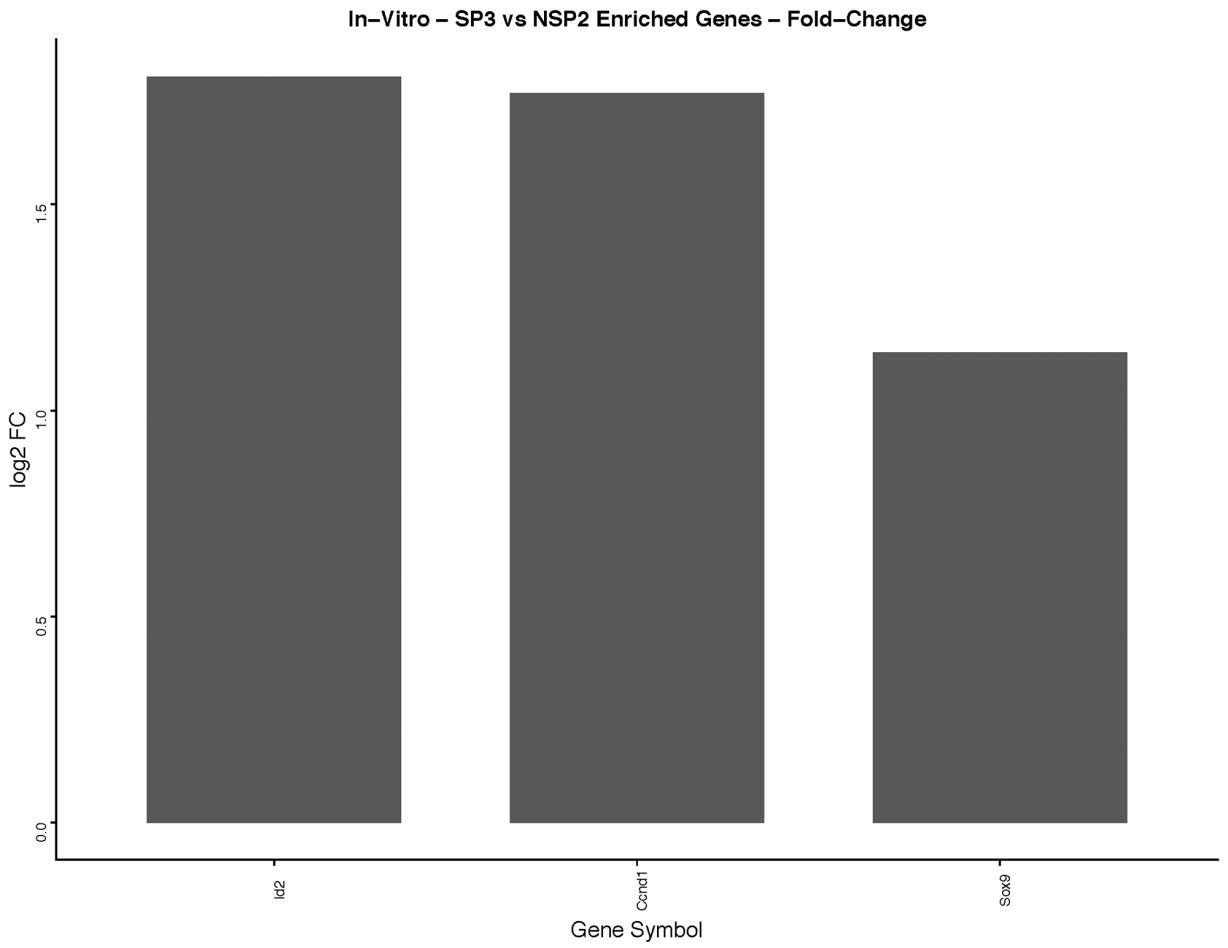
*In-vitro* analysis of, SP3 vs. NSP2, DEGs identified in cellular proliferation and cellular differentiation pathways GO, and KEGG pathway analysis was used to select differential expressed gene's (DEGs) identified in cellular proliferation and cellular differentiation. Differential gene expression parameter: log2|Fold Change|≥3.0.

**Figure 9:**
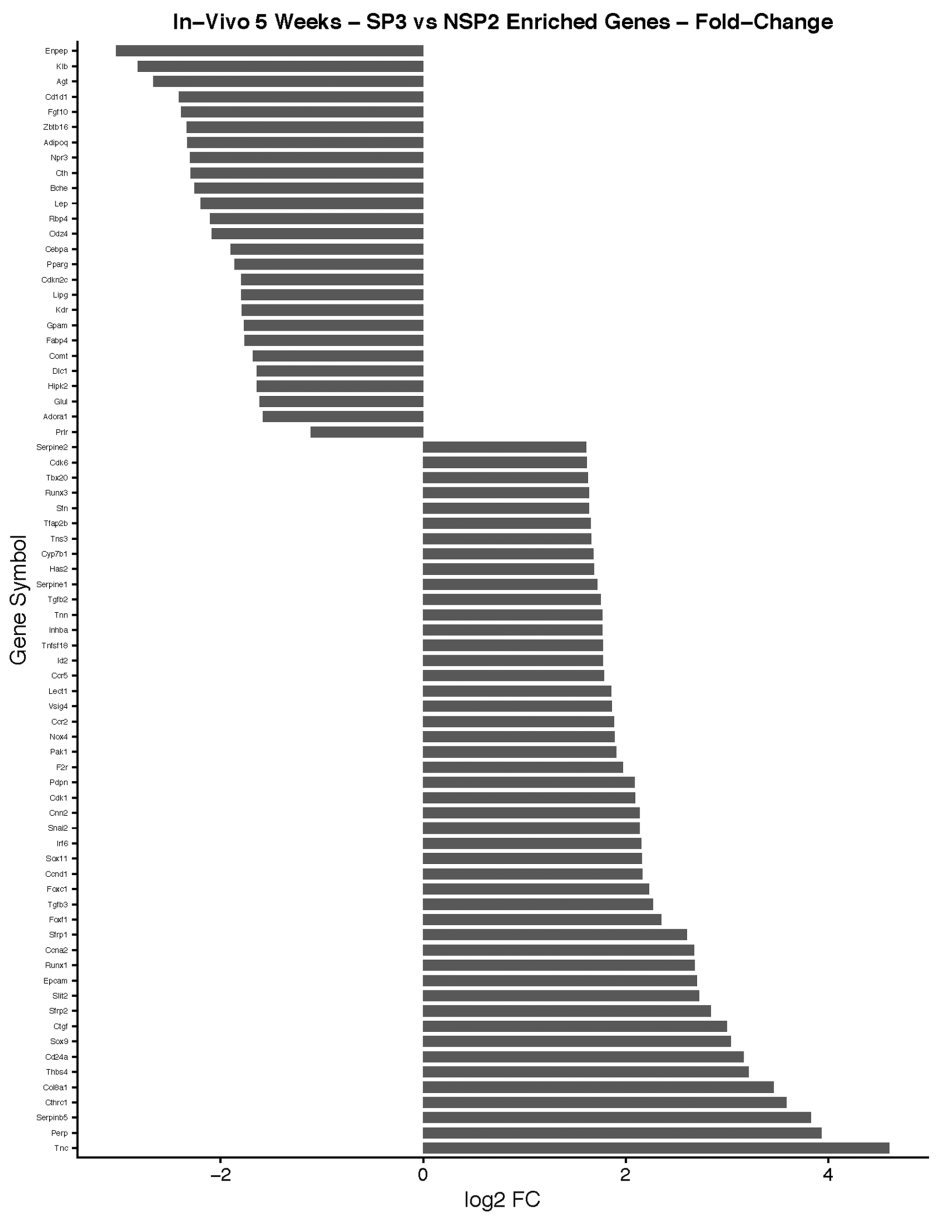
*In-vivo* 5-week analysis of, SP3 vs. NSP2, DEGs identified in cellular proliferation and cellular differentiation pathways GO, and KEGG pathway analysis was used to select differential expressed gene's (DEGs) identified in cellular proliferation and cellular differentiation. Differential gene expression parameter: log2|Fold Change|≥3.0.

**Figure 10:**
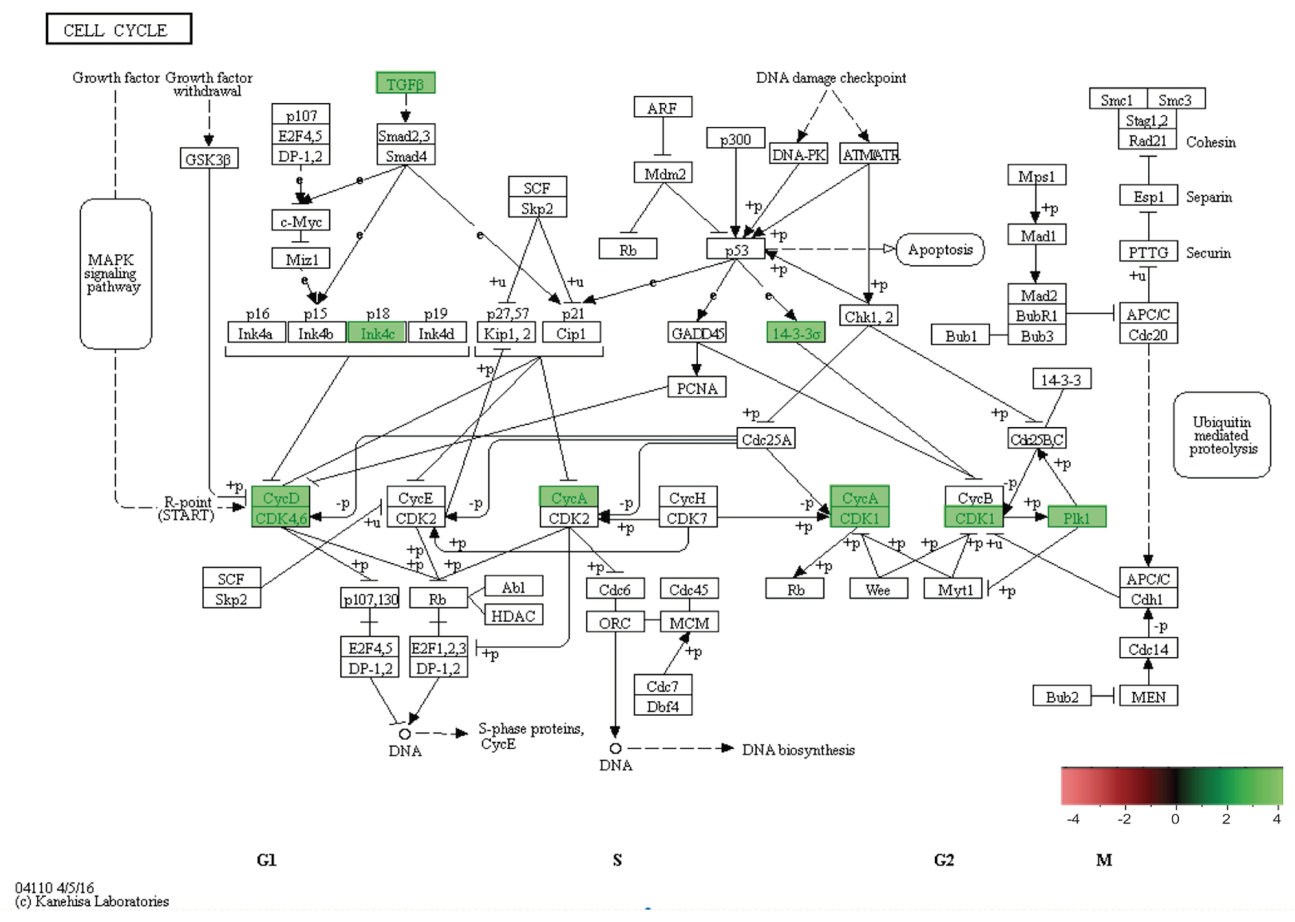
*In-vivo* 5-week KEGG pathway analysis Gene expression, for a clustered subset of genes in the cell cycle pathway, is up-regulated. The cell cycle pathway is adapted from the KEGG database. DEGs up-regulated are in (green), and down-regulated are in (red). Differential gene expression parameter: log2|Fold Change|≥3.0.

**Figure 11:**
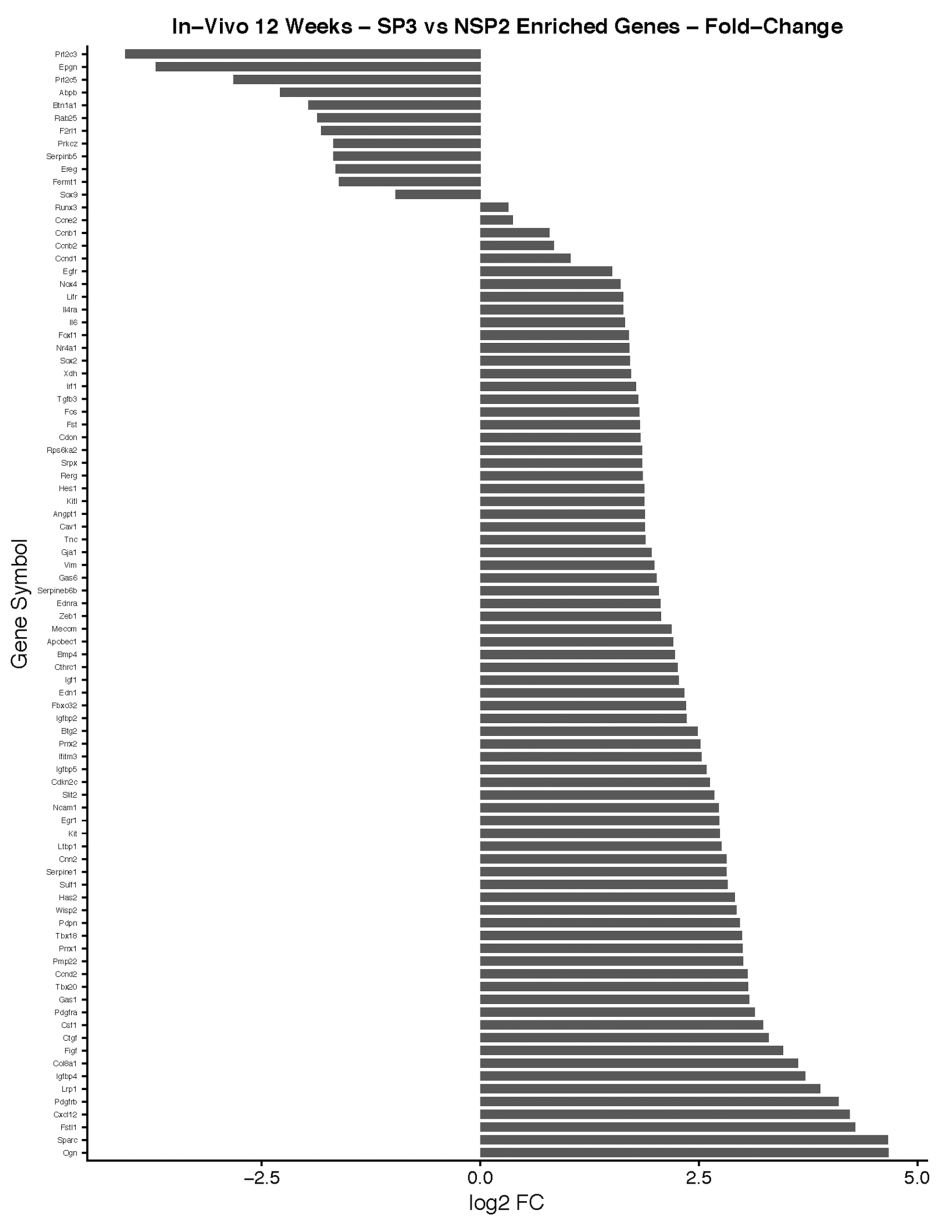
*In-vivo* 12-week analysis of, SP3 vs. NSP2, DEGs identified in cellular proliferation and cellular differentiation pathways GO, and KEGG pathway analysis was used to select differential expressed gene's (DEGs) identified in cellular proliferation and cellular differentiation. Differential gene expression parameter: log2|Fold Change|≥3.0.

**Figure 12:**
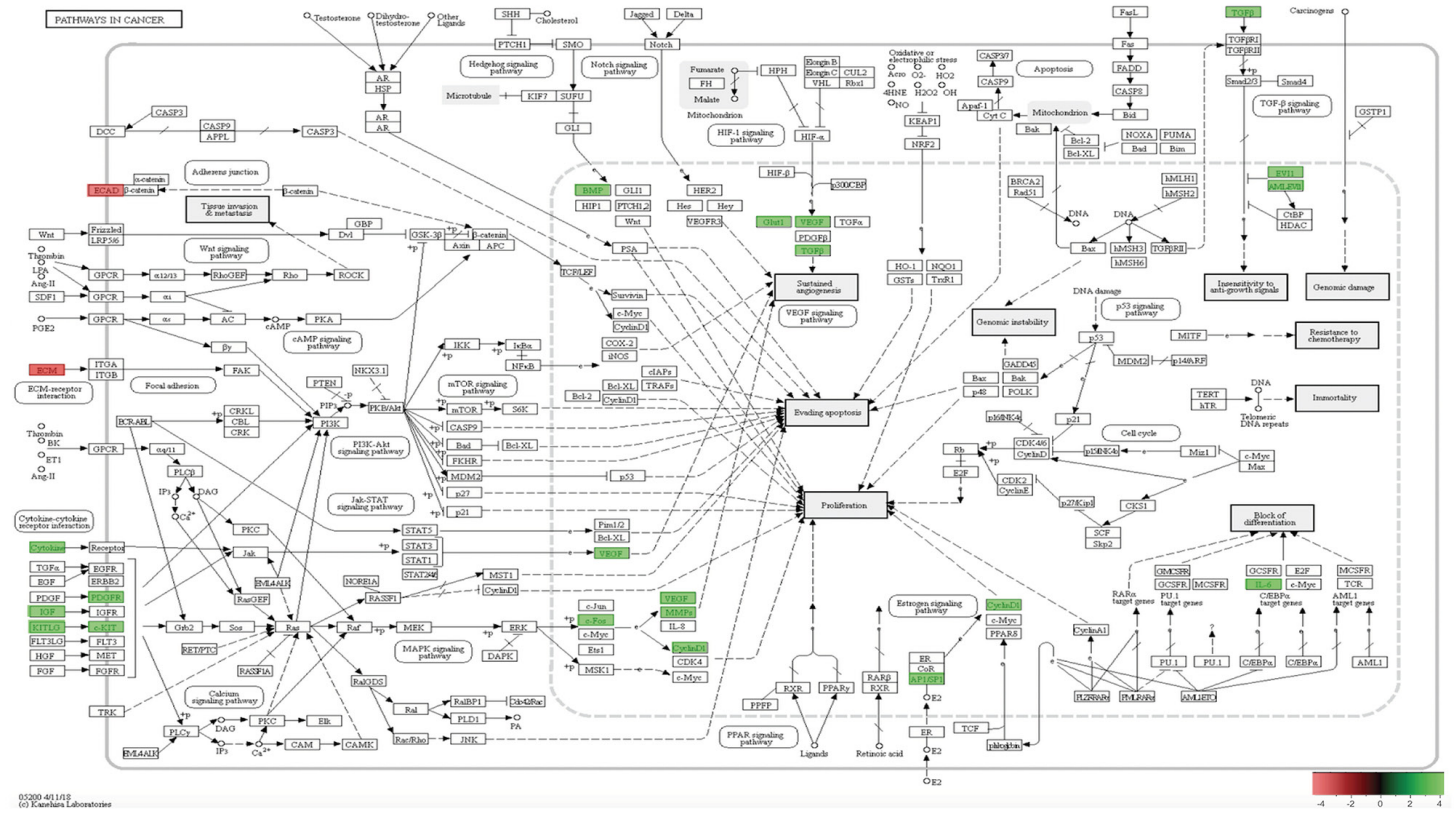
*In-vivo* 12-week KEGG pathway analysis Gene expression, for the clustered subset of genes in pathways in cancer, is up-regulated. The pathways in cancer are adapted from the KEGG database. DEGs up-regulated are in (green), and down-regulated are in (red). Differential gene expression parameter: log2|Fold Change|≥3.0.

GO enrichment software analysis distributed significantly DEGs into biological process, molecular function and cellular component categories. We selected a sub-category of the biological process that indicated significantly DEGs associated with cellular proliferation and mammary gland development. *In-vitro* GO enrichment analysis indicated 3 DEGs associated with cellular proliferation and gland development (Figure 8). *In-vivo* 5-week, GO enrichment analysis indicated 71 DEGs associated with cellular proliferation (Figure 9). KEGG pathway enrichment identified 9 DEGs, of the 71 analyzed by GO enrichment, clustered in the cell cycling pathway (Figure 10). *In-vivo* 12-week, GO enrichment analysis indicated 72 DEGs associated with cellular proliferation (Figure 11). KEGG pathway enrichment identified 23 DEGs, of the 72 analyzed by GO enrichment, clustered in the: Adherens Junction; ECM-receptor interaction; Cytokine-Cytokine receptor; PPAR signaling pathway; Estrogen signaling pathway; HIF-1alpha signaling pathway; TGF-B signaling pathway; and the Hedgehog signaling pathway (Figure 12).

### Validation of microarray data via Western blot analysis

4 genes, derived from GO enrichment categories: cellular proliferation and gland development, were chosen randomly for validation. Validation results indicated the presence of protein expression concordant with microarray RNA expression, for *in-vivo* 5-weeks (Supplementary Figure 1). We show that NSP2 (lobular incompetent) progenitor cells have similar protein expression to Balb/C (Control), as opposed to SP3 (lobular competent) cells. SP3 protein expression for ID2, Sox9, Cytokeratin 5, Cytokeratin 8 and Internal control beta-Actin, reveals apparent attenuation, in band intensity, compared to Balb/C and NSP2. This is expected because SP3 grows to fill less of the mammary fat pad than NSP2 or Balb/C [13, 14].

## DISCUSSION

Previous studies have demonstrated the importance and roles that distinct stem/progenitor cells, play in the development of many types of cancer, including breast cancer [21]. These studies postulate that understanding molecular and signaling pathways that are responsible for the self-renewal and survival of these cells are essential to understanding the mechanisms involved in the heterogeneity observed among subtypes of human breast cancer [22, 23, 24]. Further characterization of those distinct progenitors is necessary for testing that hypothesis. Using Comma-Dβ and clonal derivatives such as SP3 (lobular competent progenitor) and NSP2 (lobular incompetent progenitor) has proven to be advantageous models to help elucidate underlying mechanisms essential to tumorigenesis and pathways necessary for cell fate differentiation in the mammary gland.

### Cyclin D1, Id2, and Sox9 expression are essential for SP3 lobular genesis, *in-vitro*, via Gene Ontology (GO) enrichment analysis

Kittrell and colleagues investigated and confirmed Comma-Dβ clonal derivatives possessed different outgrowth potential and characteristics [13]. To date, SP3 and NSP2 are the only clonal derivatives that show restricted progenitor, lobular competency and lobular incompetence, respectively. Using our DEG screening algorithm (Figure 3) and GO enrichment analysis, we show a total of 45 DEGs overexpressed, ≥3.0 fold, in SP3 vs. NSP2 (Table 1). Of the 45 DEGs, CCND1 (Cyclin D1), Id2 (Inhibitor of DNA binding 2) and Sox9 (SRY-Box 9) (Figure 8), were overexpressed in SP3 than NSP2. In addition, associated with biological processes such as: Mammary gland alveolus development; response to estradiol; regulation of G1/S transition of cell cycle; and mammary gland epithelial cellular proliferation, *in-vitro* (Figure 5A). This data in combination with Behbods’ findings, suggests that SP3 are in a state of readiness to enter the cell cycle and overexpression of cell cycling genes may be involved in tumorigenesis [25]. NSP2 lack of expression of Cyclin D1, Id2 and Sox9 equally suggest cellular proliferation and differentiation is tightly regulated for ductal morphogenesis. Cyclin D1 and Id2 activity has been well established for being critical in the self-renewal and differentiation of mammary progenitors as their ablation leads to a failure to maintain the mammary epithelial regenerative potential, defects in luminal lineage differentiation and tumorigenesis. Although Cyclin D1 and Id2 have been purported to regulate a myriad of cellular proliferative pathways such as the RANKL-RANK-Id2-p21 and PR-IGF2-Cyclin-D1-p27 pathway [26]. Wang and colleagues demonstrated Id2 regulation on the proliferation of squamous cell carcinoma, *in-vitro*, via the NF-κB/CyclinD1 pathway. Therefore, it is possible that SP3 lobular competency is under control by a synergistic regulatory relationship of Id2 and Cyclin D1 [27].

Further investigation would be necessary to elucidate the possible interplay of Id2 and CyclinD1 in SP3 lobular genesis potential. We also show Sox9 overexpressed in SP3 vs. NSP2. It is unclear if these genes work in concert with each other; however, Sox9 is essential for mammary gland development, and it has been shown that silencing Sox9 reduced cellular proliferation and invasion, in breast cancer cell lines [28].

### KEGG pathway analysis illustrates evasion of growth suppressors in cell cycle thus may lead to tumorigenesis in SP3, 5-week *in-vivo*

Mammary gland development is essentially postnatal. At the onset of puberty, approximately 5-weeks, large bulbous structures (lobules), the terminal end buds, develop at the distal ends of the mammary duct. During this process, the terminal end buds and their subtending ducts branch and progress rapidly until they reach the limits of the mammary fat pad [29]. The Comma-Dβ cell line is a unique model, to understand underlying self-renewal mechanisms, due to its ability to form outgrowths composed of branched ducts and alveolus-like structures reminiscent of normal mouse mammary gland, however, also form tumors over time, *in-vivo* [29, 13]. Here we present global transcriptome data of Comma-Dβ clonal derivative’ SP3 and NSP2, during the active regenerative growth phase.

In combination with our *in-vitro* transcriptome analysis of SP3 vs. NSP2, we show that there is a persistent overexpression of Cyclin D1 (Ccnd1), Inhibitor of DNA binding 2 (Id2) and SRY-Box9 (Sox9), *in-vivo* at 5 weeks (Figure 9). This suggests, irrespective of the mammary microenvironment signaling contribution, Cyclin D1, Id2, and Sox9 overexpression is essential to SP3 epithelial cellular proliferation. Utilizing KEGG pathway analysis, we show 9 DEGs: Transforming Growth Factor beta 2 (TGFβ2); Transforming Growth Factor beta 3 (TGFβ3); Cyclin-dependent kinase 4 inhibitor C (p18); Cyclin D1; Cyclin-Dependent Kinase 6 (CDK6); Cyclin A2 (CCNA2); Polo-like kinase 1 (Plk1); Cyclin-Dependent Kinase 1 (CDK1); and Stratifin (Sfn), clustered and overexpressed greater than 3.0-fold, in SP3's cell cycle pathway (Figure 10). Figure 10 illustrates, under normal conditions, cellular stress (e.g., U.V radiation) leads to, up-regulation of TP53 (p53), which blocks Cyclin B1 (CCNB1) and Cyclin-dependent kinase 1 (CDK1) through activation of Stratifin (Sfn) and Growth Arrest DNA Damage (GADD45). p53, simultaneously, inhibits Cyclin D1 up-regulation through activation of GADD45 and Proliferating Cell Nuclear Antigen (PCNA), to arrest cell growth and proliferation. Interestingly, Comma-Dβ and its clonal derivatives, SP3 and NSP2, possess two mutations in TP53 (p53), ablating wildtype function [13]. Therefore, it's reasonable to suggest that cell cycle deregulation, is inherent in SP3, due to p53 ablation. However, when combined with the overexpression of the 9 DEGs, SP3 during 5-week rapid growth, tumorigenesis is initiated due to evasion of growth suppressors and overexpression of oncogenes. Considering, NSP2 does not show overexpression of these DEGs but still possess dysfunctional p53 activity suggest a need of combinative dysregulation of those 9 DEGs to initiate tumorigenesis.

Kittrell and colleagues discovered, after transplantation, NSP2 forms outgrowths that fill the cleared mammary fat pad and does not differentiate (form secretory lobules) when exposed to pregnancy hormones. In addition, SP3 cells typically fill 20% to 59% of the mammary fat pad (Kittrell et al., unpublished data). Utilizing Western blot analysis, to validate expression profiles of selected cell lines, we show apparent attenuation, of 4 random DEGs: ID2, Sox 9, Cytokeratin 5 and Cytokeratin 8 band intensity, in SP3 compared to NSP2 and Balb/C. The attenuation of band intensity is also prevalent in beta-Actin, which is contributed by stromal cells as well as epithelium, *in-vivo* (Supplementary Figure 1). Accordingly, this data validates our expression profile array, qualitatively.

### SP3 DEGs, in multiple carcinogenic pathways, is essential for tumorigenesis and metastasis, *in-vivo* 12-weeks

By 10-12 weeks of age, the terminal end buds regress to form blunt-ended ducts, and the gland settles into its adult cycle and enter maintenance phase until initiation of next reproductive cycle [29]. During the maintenance phase, secretory epithelium undergoes massive apoptosis, and the gland is remodeled to a similar virgin female. However, our data suggest, similar to *in-vivo* 5-weeks, SP3 overtime, evade cell cycling checkpoints and shows a myriad of tumorigenic pathways up-regulated, *in-vivo* 12-weeks.

Utilizing GO and KEGG pathway enrichment analysis (Figure 12), our data shows Cadherin 1 (Cdh1) and Laminin, gamma 2 (Lamc2), down-regulation, is associated with abnormal Adheren junction and Focal adhesion extracellular matrix (ECM) responses. Consequently, ECM proteins have an immense effect on stromal cells in tumor tissue. For example, different rotational movement observed during normal 3D morphogenesis, regulates laminin matrix assembly and is lost in cancer derived epithelial cells [30]. This suggest, during SP3 remodeling maintenance phase, ECM proteins may have adverse effects, *in-vivo*, which may play a role in tumor initiation. Interestingly, five transducing signaling pathways were identified as essential modifiers of SP3 maintenance *in-vivo* 12-weeks. One of the critical transducing signaling pathways identified was the Mitogen-activated protein kinase (MAPK) signaling pathway. DEGs responsible for this pathways’ up-regulation were: Platelet-derived growth factor receptor, beta and alpha polypeptide (Pdgfrb and Pdgfra); Kit ligand (Kitl); Insulin growth factor (Igf1); Kit oncogene (Kit) c-Fos induced growth factor (Figf); Matrix metallopeptidase 2 (Mmp2). The MAPK cascades transmit and amplify signals involved in cell proliferation and are a key molecule in the regulation of breast carcinogenesis [31]. Signal transducing pathways, such as MAPK, serves as an indicator of the intensity of signal cross-talking induced by various growth factors, steroid hormones, and G-protein receptor-mediated ligand receptors. Hence, overexpression of growth factors such as Igf1, Kit, Kitl, Pdgfra, and Pdgfrb, increases signal transduction intensity and may add to SP3 propensity for cellular proliferation. However, our data suggest, the Hedgehog and HIF-1 (Hypoxia Inducible Factor) signaling pathway, may be crucial for invasion and metastasis. We show via GO and KEGG pathway analysis, overexpression of, Bone Morphogenic Protein 4 (BMP4), in the Hedgehog pathway, signals increased cellular proliferation while Glucose transporter 1 (Glut1); Vascular Endothelial Growth Factor (VEGF) and Transforming growth factor, beta 3 (Tgfb3), overexpression, in the HIF-1 signaling pathway, lead to sustained angiogenesis [32, 33]. Interestingly, we also show Cyclin D1 and Activator Protein 1 (AP1) via GO and KEGG pathway analysis, overexpression, to be associated with altered estrogen signaling pathway regulation [34] and MDS1/ EVI1 complex locus (Mecom), overexpression, able to modify cellular insensitivity to anti-growth signals, in tumor initiation [35]. Lastly, Unlike SP3 *in-vitro* and *in-vivo* 5-week analysis, Id2 and Sox9 were under expressed.

Our data suggests that overexpression of DEGs, in SP3, leads to abnormal ECM (adheren junction and focal adhesion); increased activity of MAPK, Hedgehog, HIF-1, Estrogen signaling and TGF beta, which is essential for tumorigenesis, *in-vivo* 12 weeks. These DEGs should be investigated further due to possible therapeutic strategies that could be engendered.

## MATERIALS AND METHODS

### Immunohistochemistry

Fifty thousand NSP2 cells were mixed with an equal number of normal mammary epithelial cells (MEC) from Balb/C mice and inoculated into epithelium-cleared mammary fat pads of Nu/Nu females in 10 μl of DMEM without serum. The hosts were bred and allowed to reach parturition. The inoculated fat pads were removed, fixed in 4% paraformaldehyde for 1 hour, permeablized and reacted with complete X-gal substrate as described earlier [15]. The NSP2 structures were distinguished from the MEC by the presence of beta-galactosidase enzymatic activity. Subsequently, the outgrowths were dehydrated in a series of alcohols and embedded in paraffin. Six-micron sections were cut, placed on slides reacted with antibodies against total caseins [16], RANKL (Abcam-cat#9957)L. Progesterone receptor (PR-DAKO-PgR636), estrogen receptor alpha (ERα-Santa Cruz-sc542) at dilutions of 1:1000, 1:150, 1:200 and 1:75, respectively.

### Sample preparation

CDβ, SP3, NSP2 and normal Balb/C MEC were cultured in identical tissue culture medium to provide *in-vitro* cell samples. 20,000 cells from each of these cultures were injected in 10ml of DMEM without serum into the epithelium-cleared inguinal mammary fat pads of Nu/Nu 3-week-old females three samples were collected from cultures and 5 weeks and 12 weeks after inoculation into Nu/Nu mammary fat pads.

### RNA isolation from cultured cells

Prior to working with RNA, surfaces and instruments were sprayed with RNase Zap (Ambion-AM9780). RNA was isolated from cultured cells according to the RNeasy Mini protocol (Qiagen 7414). Cells were washed in PBS, then lysed with buffer RLT and 10 μl/ml β-mercaptoethanol, using 350 μl per T25 flask or 600 μl per 10 cm plate. The lysate was collected with a cell scraper, placed into an RNase-free tube, and homogenized by applying it to a QIAshredder spin column (Qiagen 79654) and spinning for 2 min at 13 rpm. The homogenized lysate could be stored at -80°C or used for the RNA isolation. The RNeasy Mini protocol was followed, including on-column incubation with RNase-free DNase (Qiagen 79254). After the appropriate washes, RNA was eluted with 30 μl RNase-free water, and the concentration was determined using a Nanodrop spectrophotometer. RNA was stored at -80°C. Each sample was isolated and used individually, in triplicate.

### RNA isolation from tissues

All surfaces and instruments were cleaned with RNase Zap. RNAlater-ICE (Ambion AM7030) was chilled in polypropylene tubes at -80°C or on dry ice. Each mouse was individually euthanized and sprayed with RNase Zap before quickly removing mammary tissue and placing it directly into liquid nitrogen. Frozen tissues were then soaked in chilled RNAlater-ICE at -20°C for at least 16 hr to enable complete penetration. Tissues were weighed and cut to ≤100 mg. RNA was isolated according to the RNeasy Lipid Tissue Kit Mini protocol (Qiagen 74804), including on-column DNase treatment. RNA was eluted with 30 μl RNase-free water, and the concentration was determined using a Nanodrop spectrophotometer. RNA was stored at -80°C. Each sample was isolated and used individually, in triplicate.

### RNA quality control

Immediately prior to cDNA synthesis, the purity and concentration of RNA samples were determined from OD_260/280_ readings using a dual beam UV spectrophotometer and RNA integrity was determined by capillary electrophoresis using the RNA 6000 Nano Lab-on-a-Chip kit and the Bioanalyzer 2100 (Agilent Technologies, Santa Clara, CA) as per the manufacturer's instructions.

### cRNA synthesis and labeling

Total RNA was processed by the random primed RT-IVT-RT method using the GeneChip WT cDNA Synthesis and Amplification Kit (Affymetrix, Santa Clara CA), according to the manufacturer's instructions. In brief, first and second strand cDNA was synthesized from 200 ng of total RNA by reverse transcription using T7 promoter-(N_6_) oligonucleotides as primers, cRNA was then synthesized by *in-vitro* transcription, and amplified single-stranded cDNA was synthesized by reverse transcription using random hexamers as primers. Amplified cDNA was fragmented and labeled with biotin using the GeneChip WT Terminal Labeling Kit (Affymetrix, Santa Clara CA), according to the manufacturer's instructions. In brief, cDNA was glycosylated by UDG, fragmented by APE1, and end-labeled by terminal transferase using a biotinylated dCTP derivative.

### Oligonucleotide array hybridization and analysis

Fragmented cDNA was hybridized for 17hr at 45°C to GeneChip^®^ MoGene 1.0 ST Arrays (Affymetrix, Santa Clara CA). The MoGene 1.0 ST arrays contain oligonucleotide probe sets that are specifically designed to interrogate and detect more than 28,000 coding and 7,000 non-coding gene transcripts (including ~2,000 long intergenic non-coding transcripts). Arrays were washed and stained using a Fluidics Station 450 (Affymetrix) according to the manufacturer's recommended procedures. The arrays were stained with phycoerythrin-conjugated streptavidin (Invitrogen, Carlsbad, CA) and the fluorescence intensities were determined using a GCS 3000 7G high-resolution confocal laser scanner and analyzed using programs resident in the Affymetrix GeneChip Expression Console (Affymetrix).

### Data analysis

Expression data were analyzed following background correction, quantile normalization, and probe set signal summarization by RMA in Affymetrix Expression Console [17, 18]. RMA Quality Control outputs from Expression Console were used to identify potential outlier arrays; outlier evaluation was also performed by Principal Components Analysis in GeneMaths XT (Applied Maths, Austin TX). Probe sets exhibiting significant differential expression were selected using RMA absolute Signal log ratios ≥ 1.5875 (absolute fold-change ≥ 3.0), and false-discovery rate (FDR) adjusted ANOVA p-value ≤ 0.01 and FDR-adjusted *t*-test p-value ≤ 0.01 (for pair-wise comparisons); all p-values were subjected to FDR-adjustment via the Benjamini-Hochberg step-up procedure [19]. Unsupervised hierarchical clustering and heat map generation was performed in GeneMaths XT using row mean centered log2 transformed RMA signal values. Probe set clustering was performed by the UPGMA method (Unweighted Pair Group Method using Arithmetic averages) using Pearson correlation as the similarity metric; sample clustering was performed by UPGMA method using Pearson correlation as the similarity metric.

Gene annotations, gene ontology information and biochemical pathway information were obtained from the National Center for Biotechnology Information (https://www.ncbi.nlm.nih.gov), NetAffx (https://www.affymetrix.com), the Gene Ontology (GO) Consortium (http://amigo.geneontology.org), the Kyoto Encyclopedia of Genes and Genomes (https://www.genome.jp/kegg), WebGestalt (http://www.webgestalt.org).

To investigate group-specific pathway enrichment, lists of differentially expressed MoGene 1.0 ST probe sets, or DEGs (and fold-change values), were submitted to WebGestalt and pathway enrichment against GO, KEGG, Wikipathways, and Pathway Commons databases were determined (http://www.webgestalt.org) [20]. When submitting DEGs to WebGestalt, categories/pathways involving ≤ 2 genes were discarded, and categories/pathways were only considered significantly enriched if FDR-adjusted hypergeometric test p-value ≤ 0.05. Microarray expression profile data was published publicly using NCBI Gene Expression Omnibus (GEO) public repository database (accession number: GSE121499).

### Western blot analysis

Total protein was isolated from frozen, Balb/C, SP3 and NSP2, mammary epithelial cells and homogenized using the Millipore extraction kit (Millipore Corporation, Billerica, MA). Proteins (30 and 50μg) were separated using 10% or 16% pre-cast Tris-Glycine gels and dry-transferred for seven minutes using the iBlot machine (Invitrogen, Gaithersburg, MD) onto PVDF membranes (Invitrogen, Gaithersburg, MD). The membrane was blocked using WesternBreeze Chemiluminescent Immunodetection Kit (Invitrogen) and probed with anti-ID2 (Abcam-ab85990), SOX9 (Abcam-ab3697), cytokeratin 5 (Abcam-ab53121), and cytokeratin 8 (Abcam-ab154301) (1:500 diluted in manufacturer primary antibody diluent buffer) overnight at 4°C. The additional probing with beta-actin (Chemicon) antibody used as an internal control. After washing with Invitrogen buffer wash (Invitrogen), the blots were treated with either Invitrogen Alk-Phos conjugated (anti-Mouse) or (anti-rabbit) for 30 minutes and washed several times. Proteins were detected by the enhanced chemiluminescence system (Invitrogen). Western analysis was performed on total cell lysate.

## CONCLUSIONS

Comma-Dβ is a unique cell line due to its ability, after transplantation, to form alveolar-like and ductal branches reminiscent of normal mouse mammary gland. Utilization of the stable Comma-Dβ’ clonal derivatives, SP3 and NSP2, provides a valuable tool for molecular characterization. It also aids in identification of self-renewal pathways and signaling pathways that are misregulated during tumorigenesis, ultimately leading to tumor heterogeneity. Here we provide data of SP3 and NSP2’s, alveolar and ductal restrictive activities, respectively, via global transcriptome analysis, *in-vitro*, *in-vivo* 5-weeks (regenerative growth phase) and *in-vivo* 12 weeks (maintenance growth phase). Our findings describe, key DEGs, such as Cyclin D1, Id2, and Sox9 to be overexpressed and essential for lobular genesis in contrast to being important for ductal morphogenesis in NSP2. In addition, we show an increased dysregulation of transduction signaling pathways, during different growth phases of SP3 vs. NSP2 development, suggesting targeting specific DEGs during different growth periods may lend to better carcinogenic therapeutic strategies in mammary tumors.

## SUPPLEMENTARY MATERIALS FIGURE



## Abbreviations

SP: side population
NSP: non-side population
DEG: differentially expressed genes
GO: gene ontology
KEGG: Kyoto encyclopedia of genes and genome
FACS: fluorescence-activated cell sorter (flow cytometry)
CDβ: Comma-Dβ
MEC: mammary epithelial cell
ECM: epithelial cell matrix
FDR: false discovery rate.
