# Supplementary material for: Microarray and pathway analysis of two COMMA-Dβ derived clones reveal important differences relevant to their developmental capacity in-vivo
Source: Oncotarget. 2019 Mar 15;10(22):2118–35. doi: 10.18632/oncotarget.26655 (PMC6481333; doi:10.18632/oncotarget.26655)
Supplement: Supplementary file 1 [file oncotarget-10-2118-s001.pdf]

Microarray and pathway analysis of two COMMA-Dβ derived clones reveal important differences relevant to their developmental capacity *in-vivo*

SUPPLEMENTARY MATERIALS

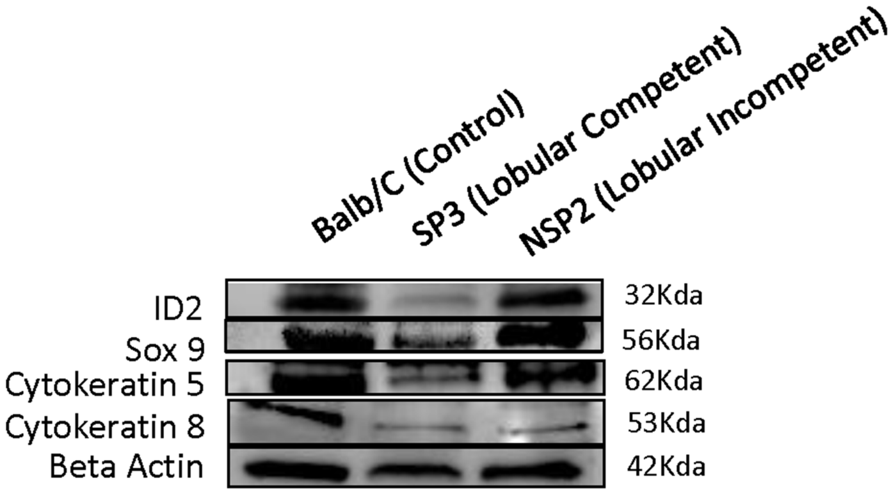

Supplementary Figure 1: *In-vivo* 5-week, western blot validation of microarray results for 4 random DEGs. ID2, Sox9, Cytokeratin 5 and 8 were probed in Balb/C (Control), SP3 (Lobular Competent), and NSP2 (Lobular Incompetent). Validation of microarray results was identified qualitatively, demonstrating the presence or absence of protein expression in samples.
